# Impact of Social Media on HPV Vaccine Knowledge and Attitudes Among Adolescents and Young Adults: A Systematic Literature Review

**DOI:** 10.3390/healthcare14010073

**Published:** 2025-12-27

**Authors:** Blessing Oluwatofunmi Apata, Anagha Hemant Tupe, Oluwabusayomi Akeju, Kelly L. Wilson

**Affiliations:** 1 Department of Health Behavior, School of Public Health, Texas A&M University, College Station, TX 77840, USA; 2 College of Nursing, Texas A&M University, Bryan, TX 77807, USA; anaghatupe@tamu.edu (A.H.T.); kwilson@tamu.edu (K.L.W.); 3 Department of Health Policy and Management, College of Integrated Health Sciences, University at Albany, State University of New York, Albany, NY 12222, USA; busayoakeju2016@gmail.com

**Keywords:** HPV vaccine, social media, adolescents and young adults, knowledge, behavior

## Abstract

**Objective**: Human Papillomavirus (HPV), a leading cause of sexually transmitted infections (STIs) and various cancers, including cervical cancer, remains prevalent in the US. Despite the HPV vaccine’s effectiveness in preventing persistent HPV infections, vaccination rates remain low. Given the significant role of social media in reaching younger populations, this systematic review examines its influence on adolescents’ and young adults (AYAs) awareness, knowledge, and attitudes toward HPV vaccination. **Methods**: Following the PRISMA guidelines, we conducted a comprehensive search across six electronic databases (ERIC, APA PsycInfo, Child Development & Adolescent Studies, CINAHL Ultimate, MEDLINE Ultimate, and PubMed) from 2011 to 2024. Empirical studies that examined the association between social media use and HPV were included. Data extraction captured the study’s purpose, design, population, outcome measures, and key results. **Results**: Seven studies satisfied the review’s inclusion criteria. Our findings reveal mixed effects of social media on AYAs’ knowledge and vaccination intentions. Some studies indicated positive associations between social media interventions and increased vaccination knowledge and intentions, while others found no significant impact. Additionally, exposure to anti-vaccine content was linked to lower vaccination intentions, especially among individuals with lower knowledge who were more vulnerable to misinformation. Interventions incorporating interactive content and loss-framed messaging were more effective in increasing vaccine intentions. **Conclusions**: This review underscores the potential of social media to influence AYAs knowledge and perceptions regarding HPV vaccination, while also highlighting the challenges posed by misinformation. Further research is needed to optimize social media interventions and combat misinformation to improve vaccination uptake.

## 1. Introduction

Human Papillomavirus (HPV) is one of the most common Sexually Transmitted Infections (STIs). It is linked to various cancers, including cervical, head and neck, and anogenital cancers such as vulvar, penile, vaginal, and anal cancers [1,2]. In the US, 13 million individuals were newly infected with HPV in 2018, and 42 million people had HPV-related diseases, contributing to a significant public health burden [3]. HPV causes about 36,000 cases of cancer in both men and women [4]. Fortunately, the HPV vaccine is highly effective in preventing persistent infections, which can lead to cancers [5] and genital warts [6]. The Gardasil-9TM vaccine offers protection against multiple HPV types, including the high-risk types 16 and 18, which are responsible for most HPV-related cancers, as well as types 6, 11, 31, 33, 45, 52, and 58 [7].

The US Advisory Committee on Immunization Practices (ACIP) recommends HPV vaccination for individuals ages 9–26, ideally starting at ages 11–12, to maximize effectiveness before HPV exposure [8]. Two doses are recommended if the series is started before age 15, while three doses are recommended for individuals aged 15 or older [9]. However, despite these recommendations, HPV vaccination rates remain relatively low in the US. Lee et al. [10] found that only 46% of adolescents initiated the HPV vaccine and 36% completed the series. While data from the National Immunization Survey-Teen (NIS-Teen) show improvements in vaccination rates, with 76.9% of 13–17-year-olds initiating the HPV vaccination series in 2021, completion rates remain lower than those for other vaccines, such as Tdap and meningococcal [11].

Several factors contribute to low vaccination rates, including parental concerns about vaccine safety, limited knowledge, and adolescents’ interactions with the healthcare system [12]. A growing concern is the role of misinformation on social media platforms, which can influence vaccine hesitancy, particularly among adolescents [13,14]. As social media use among teens continues to rise, platforms such as TikTok, Instagram, Facebook, and X (formerly Twitter) have become major sources of health information [15,16]. Adolescents are avid social media users, and a significant proportion of teenagers in the US engage with social media. Research indicates that 9 out of 10 teens use YouTube, and platforms like TikTok, Snapchat, and Instagram remain popular among adolescents [17]. Furthermore, 96% of teenagers in the US use the internet daily, with a significant increase in the number of teens who are constantly online [18].

While most HPV vaccination efforts focus on parents or healthcare providers, adolescents’ and young adults’ knowledge and attitudes toward the vaccine also play a crucial role in initiating and completing vaccination. Young adults, particularly those aged 18–26, represent a key catch-up population who may have missed vaccination in adolescence but remain at risk of HPV infection and HPV-related cancers [19]. Including young adults in HPV vaccination research provides valuable insight into this transitional developmental stage, where independent health decision-making, sexual debut, and evolving digital information consumption converge. Moreover, social media engagement tends to increase during late adolescence and early adulthood, making this age group particularly susceptible to both accurate information and misinformation about vaccines [19]. Therefore, the goal of this review is to examine how social media influences adolescents’ and young adults’ awareness, knowledge, and attitudes regarding the HPV vaccine, to identify evidence-based strategies to improve vaccine uptake across the entire vaccination-eligible population.

## 2. Materials and Methods

We followed the Preferred Reporting Items for Systematic Reviews and Meta-Analyses (PRISMA) guidelines [20] to systematically examine peer-reviewed literature on how social media use affects HPV vaccination. The search for scholarly peer-reviewed articles published in English was conducted on 18 June 2024, across six prominent electronic databases: ERIC, APA PsycInfo, Child Development & Adolescent Studies, CINAHL Ultimate, MEDLINE Ultimate, and PubMed. The search was limited to studies published from 2011 through June 2024. The search terms used for each database are listed in Table 1.

We included empirical studies that measured both social media use and HPV vaccination among adolescents and young adults (AYAs) aged 10–26 years in the US. Studies were excluded if they focused solely on adults or if they measured social media use and HPV vaccination without specifying the connection between the two. Additionally, studies that recruited participants via social media but did not actively measure social media use were excluded.

Covidence, a Cochrane technology platform, was used to manage the review process [21]. All retrieved articles were imported into Covidence, where duplicates were automatically removed. Two reviewers independently screened all titles and abstracts for eligibility, and eligible articles proceeded to full-text review. Conflicts were resolved through discussion during reviewer consensus meetings. The inter-rater reliability for the full-text screening phase was high, with a Cohen’s kappa score of 0.875.

**Figure 1:**
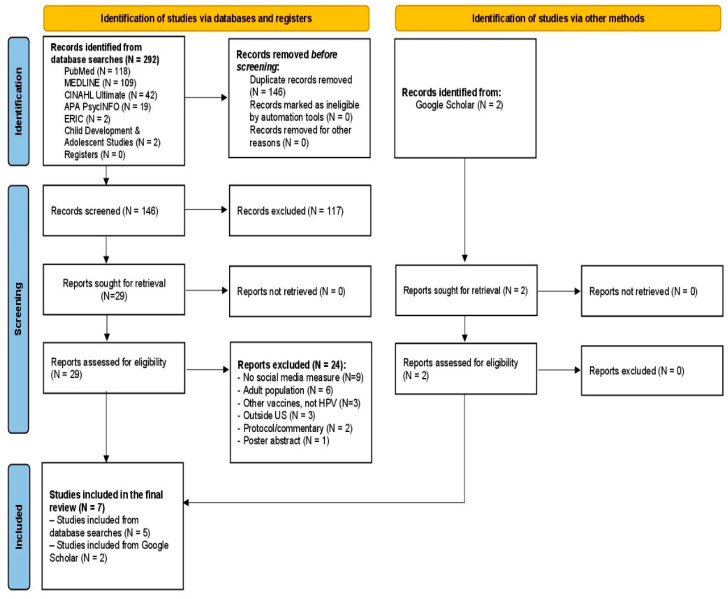
PRISMA flow diagram for the included studies [20].

A total of 294 articles were retrieved: 292 studies from the six databases and two manually added. After removing 146 duplicates upon import to Covidence, the titles and abstracts of the remaining 148 studies were screened for eligibility. Of these, 117 were excluded for various reasons. Specifically, six studies were excluded because they involved only adult samples. Other studies were excluded either for describing sample recruitment via social media without measuring social media use or for failing to measure the connection between social media and HPV vaccination. Ultimately, seven studies met the inclusion criteria and were included in this review. Data from the included studies were extracted by one reviewer and verified by a second reviewer. The PRISMA diagram of the review is shown in Figure 1.

Of the seven included studies, five (71%) [22,23,24,25,26] used a randomized experimental design, while two [27,28] (29%) used a non-randomized design. Also, only two [22,23,24,25,26,27,28] (29%) focused on adolescents (aged 10–19 years), while five [23,24,25,26,27] examined populations of young adults (aged 20–26 years) within the HPV vaccination catch-up age range. Findings were synthesized collectively to capture insights across this broader developmental period relevant to HPV vaccination. Details about the characteristics of the studies are provided in Table 2.

A thematic analysis was conducted to identify and synthesize patterns across the included studies. The analysis focused on four key outcomes related to HPV vaccination: knowledge, perception, attitude, and behavioral intentions.

### Quality Assessment

Five studies that utilized a randomized experimental design were assessed using the RoB 2 tool [29], while the two non-randomized studies were evaluated using the ROBINS-I [30].

## 3. Results

### 3.1. Knowledge of HPV and the Role of Social Media

The reviewed literature indicates varying impacts of social media on HPV vaccine knowledge. One study demonstrated that a three-month social media health intervention improved HPV knowledge among adolescents, especially those who fully engaged with the content [22]. However, another study observed that a Twitter-based intervention had no statistically significant effect on HPV knowledge scores (56% vs. 57%, *p* = 0.858) [27].

### 3.2. Perception of HPV and Vaccine Efficacy

Perceptions of the HPV vaccine were shown to be influenced by the type of media exposure. Nan and Daily [23] found that exposure to mixed blogs polarized participants’ perceived efficacy of the HPV vaccine. Those who already believed in vaccine efficacy saw their confidence increase (b = 0.473), while those with doubts experienced a slight reduction in their perceived vaccine efficacy (b = 0.036). Similarly, Kim et al. [24] noted that exposure to credible misinformation correction content reduced HPV misperceptions, while exposure to misinformation increased them. Additionally, media channels significantly affected perceptions of HPV severity and vaccination barriers. Participants exposed to online newspapers perceived greater severity of HPV-related risks compared to those exposed to Facebook (M = 5.08 vs. 4.63, *p* < 0.05) [25].

### 3.3. Attitudes and Behavioral Intentions Toward HPV Vaccination

Mohanty et al. [28] observed that advertising campaigns alone had a minimal effect on HPV vaccine uptake, even when common barriers such as parental consent and cost were minimized. Ortiz et al. [22] noted that, despite knowledge gains from a Facebook intervention, vaccination rates after 6 months were not significantly influenced. In terms of behavioral intentions, Leader et al. [26] found a positive relationship between social media engagement and vaccination intentions (r = 0.43, *p* = 0.01), highlighting the role of active participation in shaping intent. However, Lee and Cho [25] found no significant difference between newspaper and Facebook in their overall effects on behavioral intentions to vaccinate (*p* = 0.94), although loss-framed messages, that is, messages that emphasized the consequences of not vaccinating were more effective than gain-framed messages which emphasized the benefits in increasing vaccination intentions (M = 4.90, SD = 1.33). In contrast, Allen et al. [27] found that a Twitter campaign did not significantly change the vaccination intention at six (*p* = 1.000) or 12 months (*p* = 0.617) among those who had not yet started or completed vaccination

### 3.4. Risk of Bias Across Studies

Overall, the RoB 2 trials demonstrated strong methodological quality, with three studies [23,24,25] rated as low risk, one [26] as having some concerns, and one [22] as high risk. The high-risk judgment originated from substantial deviations from intended interventions and missing outcome data.

In contrast, the two non-randomized studies [27,28] showed a markedly higher risk of bias. Both exhibited a serious to critical risk of confounding because key variables were not controlled, and no adequate adjustment strategies were employed. Although outcome measurement was consistent and low risk across both studies, concerns persisted related to selection processes, missing data, and the absence of pre-specified analysis plans. These limitations justify interpreting findings from the non-randomized studies with caution.

Overall, the evidence base demonstrates that randomized studies provide the most reliable insights, while the non-randomized studies offer valuable contextual information but are limited by methodological constraints.

## 4. Discussion

This study emphasizes the role of social media in shaping knowledge about HPV, showing that increased interaction with accurate and informative online content can improve understanding of the vaccine. One of the included studies, which evaluated a Facebook-based intervention, supports this by indicating that social media use can lead to higher HPV knowledge [22]. However, another study using a Twitter intervention found no significant link between social media engagement and HPV-related knowledge [27]. This mixed evidence highlights the need for more research to clarify how social media influences vaccine knowledge. Similar patterns have been observed in broader research, indicating that while social media use is positively associated with HPV awareness, it is only loosely associated with HPV knowledge. This suggests that mere exposure may increase awareness but does not necessarily lead to a deeper or more accurate understanding [31,32]. Notably, these studies did not analyze individual platforms. Conversely, a targeted Facebook campaign effectively boosted HPV knowledge among underrepresented populations [33]. Variations across studies may result from differences in engagement levels, intervention duration, the type of content shared, or platform-specific features. Since social media platforms vary widely in structure and communication style, future research should examine how these factors, such as differences between Facebook and Instagram, impact knowledge acquisition and related behavior change.

Findings related to perception further indicate that exposure to misinformation on social media can heighten HPV vaccine misperceptions and that individuals who already hold inaccurate beliefs are more likely to interact with misleading content [24]. This reinforces the dual nature of social media as both a tool for disseminating accurate public health information and a conduit for misinformation. Recent multi-platform analyses show that misinformation narratives about the HPV vaccine are widespread across Facebook, X (formerly Twitter), and TikTok, often recycling themes of unnecessary vaccination, adverse reactions, and mistrust of institutions. Although a greater proportion of misinformation was found on Facebook [34]. These differences likely reflect the way each platform works. Facebook’s algorithm tends to promote belief-consistent content and discussions within private or like-minded groups, allowing misinformation to circulate with limited correction. X, by contrast, is more open and fast-paced, which can spread misinformation quickly but also enables faster responses from credible sources. TikTok’s short, visually engaging videos often rely on emotional storytelling that appeals to younger users but can sometimes oversimplify or distort health information. These differences suggest that platform design and audience dynamics influence how misinformation spreads and how effectively accurate HPV vaccine messages reach users.

Prior work similarly underscores this dual role of social media, consistent with our findings that misinformation remains a major challenge [35]. Hence, efforts to leverage social media for health information should aim to counter misinformation effectively. Importantly, individuals with positive prior perceptions of vaccine efficacy are more likely to maintain accurate beliefs over time [23]. Chen et al. [36] further showed that greater knowledge reduces vulnerability to misinformation, thereby leading to more negative attitudes and lower vaccination intentions. This effect may be exacerbated by the clustering of like-minded individuals in online social networks and echoes the chamber effect, where users are selectively exposed to content that aligns with their views [37]. This dynamic can hinder the effectiveness of health campaigns in reaching those who need them most. These findings support the idea that strengthening baseline knowledge and critical evaluation skills may be essential components of any social media-based strategy to counter HPV vaccine misinformation.

Furthermore, differences in media exposure also matter. Lee and Cho [24] demonstrated that individuals exposed to Facebook perceived HPV-related risks as less severe compared to those exposed to online newspapers. Several studies suggest that Facebook’s platform structure, which emphasizes peer interaction, brevity, and algorithm-driven content, may dilute the perceived seriousness of health risks compared to the more formal, information-dense reporting typical of online newspapers. On Facebook, health messages compete with entertainment and personal content, reducing their perceived urgency or importance [32,38]. Posts may also be shorter, less detailed, and more likely to use casual language or interactive features (e.g., polls, memes), which can downplay perceived risks [33,38]. These findings underscore the value of integrating social media with traditional public health communication, as combining approaches may strengthen the overall impact of health interventions [39,40]. Social media may serve as a useful tool for initial engagement, but reinforcement through traditional methods, such as in-person communication or official health channels, is often necessary.

Regarding attitudes and behavioral intentions, our findings were mixed. Ortiz et al. [21] observed that, despite increased knowledge following a Facebook intervention, vaccination rates did not change significantly after six months. This aligns with broader research suggesting that factors beyond knowledge, such as accessibility, quality of provider recommendations, structural barriers, and social influences, play a more substantial role in shaping vaccination behavior [41,42,43]. Consistent with the Health Belief Model (HBM), this also indicates that improved knowledge may increase perceived susceptibility and benefits but may be outweighed by perceived barriers such as concerns about vaccine safety, access, and side effects, which can strongly hinder vaccination [44]. On the other hand, perceived susceptibility may be low as some believe vaccination is unnecessary if they are not sexually active [45]. Likewise, Mohanty et al. [28] found limited effects of advertising campaigns on vaccination uptake, even when common barriers such as cost were removed. These results parallel those of Allen et al. [27], who reported minimal influence of a Twitter campaign on vaccination intention. This aligns with the Theory of Planned Behavior (TPB), which posits that three key factors shape behavioral intention: attitude towards the behavior, subjective norms, and perceived behavioral control, not just knowledge. Also, a study has shown that even with good knowledge, if important referents like family, friends, or healthcare providers do not support vaccination, or if there is social stigma, individuals may not intend to vaccinate, which can be especially true in adolescent or young adult populations [46]. Together, these findings indicate that social media interventions may be more effective in shifting proximal outcomes, such as awareness and knowledge, rather than driving substantial direct changes in vaccination uptake or completion without complementary efforts to address access, clinic workflow, and social norms. Multi-component strategies that address both informational and structural barriers, such as reminders, improved access, and social support, are more likely to produce meaningful change [47,48]. Additionally, studies show that the perceived credibility of social media content plays a key role in HPV vaccine decision-making [49,50]. Thus, public health communication efforts should prioritize improving message credibility, transparency, and source verification across platforms.

Finally, our review found that high engagement with vaccine-related content on social media positively influences vaccination intentions [27]. This is supported by studies showing that engagement helps credible, pro-vaccine messages stand out in crowded, often misinformation-rich environments, enhancing their reach and persuasive power [51,52]. Social media strategies that foster dialogue, incorporate personal narratives, and encourage user interaction (through comments and shares) are particularly effective in building trust and promoting positive attitudes toward vaccination, especially in communities with high hesitancy or mistrust [53]. This highlights that merely rolling out campaigns is not enough; efforts must focus on ensuring meaningful engagement with the content to achieve desired outcomes. Message framing also plays a pivotal role. Lee and Cho [24] demonstrated that loss-framed messages, which emphasize the consequences of not vaccinating, were more effective than gain-framed messages highlighting benefits. Studies further show that personal storytelling and relatable narratives enhance perceived similarity and social norms, thereby increasing message acceptability, especially when delivered by trusted messengers, such as influencers [54,55]. These findings underscore the importance of strategically framing messages to optimize their impact on vaccination intentions.

### 4.1. Implications

Today, social media and digital technologies are easily accessible and widely used, including by adolescents. Social media has the power to educate, persuade, and entertain, but it can also misinform and misguide users. The findings from the included studies highlight the need for future studies on social media interventions to increase HPV vaccination uptake and completion in the US. Future research and interventions should stay current with evolving social media trends and platforms [21,26], ensure the information provided is reliable and credible [21,23], integrate social media with traditional educational methods [24,32,33], and feature content that is both engaging and carefully tailored [24,25].

The literature consistently supports that future interventions must adopt a comprehensive approach, incorporating traditional strategies such as provider recommendations, vaccination reminders, scheduling, and in-person communication [24,26]. Given the variety of social media platforms, interventions should be customized to fit the specific user demographics of each platform, acknowledging that vaccination intentions and status may differ between platforms [26]. Furthermore, interventions should consider the background and community context of the priority population [27]. For instance, adolescents may respond more strongly to messaging about short-term consequences of HPV, such as STIs like genital warts, rather than the long-term effects, such as cancers [27].

A major barrier to effective social media interventions is misinformation, yet these interventions can also address it. Evidence suggests that interventions incorporating credible sources and a balance of humorous and factual HPV information may be the most effective at increasing vaccination rates and combating misinformation [22,23]. Finally, digital media interventions must be engaging to users. Prioritizing this engagement has shown promising results in increasing vaccination intention [25], a crucial first step toward boosting vaccine uptake and reducing HPV infection rates.

### 4.2. Limitations

This literature review is based on only seven intervention-based studies in the US, which may not fully represent the broader landscape of social media’s role in influencing HPV vaccination. As a result, the findings may not be fully generalizable to the wider public, real-time social media conversations, or other countries.

Also, the studies resulting from our methods (inclusion and exclusion criteria) are not representative of all social media platforms (e.g., TikTok, Instagram), and the results should therefore be interpreted with caution. Overall, the focus on experimental and quasi-experimental studies limits the understanding of how social media influences HPV vaccination in natural, everyday settings. To gain a comprehensive understanding, more longitudinal, descriptive studies are needed to explore the dynamics and trends in the real-world social media environment.

Furthermore, although most of the included randomized studies had low risk of bias for randomization and outcome measurement, one trial was rated high overall due to deviations from the intended intervention and substantial missing outcome data, and several raised concerns about selective reporting in the absence of clearly pre-specified analysis plans. In contrast, the two non-randomized studies were at serious or critical risk of confounding, with limited control for prognostic factors and designs that made it difficult to distinguish intervention effects from underlying trends or external influences. Additionally, the use of different risk-of-bias tools for randomized and non-randomized studies reflects real design differences. Still, it complicates the direct comparison of methodological quality across all included studies.

Lastly, this review did not have a prospectively registered protocol in PROSPERO, which is a limitation, although the eligibility criteria and search strategy are described in detail to enhance transparency.

## 5. Conclusions

This review highlights the potential role of social media in shaping HPV-related knowledge, perceptions, and attitudes in the US. Across the included studies, social media-based interventions were effective in some cases at improving knowledge or perceptions. However, translating this into behavior change requires addressing barriers such as access to social norms or social support. At the same time, exposure to misinformation on social media was shown to contribute to misperceptions and, in some cases, decreased confidence in vaccination. Given these mixed effects, social media should be viewed as one component of a broader communication environment rather than a stand-alone solution.

Also, our study demonstrates the promise of carefully framed, tailored messaging and engaging content. While this review primarily focused on AYAs as a collective group, the findings also suggest the value of tailoring interventions to different developmental stages. For example, younger adolescents may benefit more from parent-guided or school-based social media education. In contrast, older AYAs who engage more independently online may respond better to peer-driven or influencer-based campaigns.

Future research should focus on more rigorous evaluations of social media-based strategies, including their impact on actual vaccination behavior, and examine how platform dynamics, message credibility, and user engagement shape outcomes. Continued monitoring of misinformation and evolving communication patterns will also be important for understanding how social media influences HPV-related decision-making over time, especially as we enter the era of artificial intelligence.

## Figures and Tables

**Table 1:** Search Terms.

Database	Concept	Search Term
ERIC, APA PsycInfo, Child Development & Adolescent Studies, CINAHL Ultimate, MEDLINE Ultimate	Social Media	(“social media” OR “social networking sites” OR “online platforms” OR Facebook OR Twitter OR Instagram OR TikTok OR YouTube) AND
	Adolescents and Young Adults	(adolescen* OR teen* OR youth OR “young people” OR “high school student*” OR “secondary school student*”) AND
	HPV Vaccination	(“HPV vaccin*” OR “human papillomavirus vaccin*” OR “HPV immuniz*” OR “HPV vaccinat*”)AND
	Health Behavior	(awaren* OR knowledg* OR attitud* OR percept* OR belief* OR opinion* OR understand* OR information OR education)
PUBMED		(((((“social media”[MeSH] OR “social media” OR “social networking sites” OR “online platforms” OR Facebook OR Twitter OR Instagram OR TikTok OR YouTube)) AND ((“adolescent”[MeSH] OR “youth”[MeSH] OR adolescen* OR teen* OR youth OR “young people” OR “high school student*” OR “secondary school student*”))) AND ((“papillomavirus vaccines”[MeSH] OR “HPV vaccin*” OR “human papillomavirus vaccin*” OR “HPV immuniz*” OR “HPV vaccinat*”))) AND ((“health knowledge, attitudes, practice”[MeSH] OR awaren* OR knowledg* OR attitud* OR percept* OR belief* OR opinion* OR understand* OR information OR education)))

The asterisk (*) was used as a truncation symbol to capture variations of word endings.

**Table 2:** Characteristics of included studies.

No	Author (Year)	Purpose	Design	Sample/Population	Outcome Measure	Key Result
1.	Leader et al., (2022) [26]	To test whether social media narrative engagement variations led to differences in HPV vaccine intentions.	Experimental	Social media participants between the ages of 18 and 26 who had not received the HPV vaccine (*n* = 607)	Intention	There was also a positive relationship between social media engagement and intentions to vaccinate (r = 0.43, *p* = 0.01)
2.	Mohanty et al., (2018) [28]	To assess the campaign reach, engagement, and HPV vaccine uptake among Philadelphia adolescents through the 3forME Facebook campaign	Quasi-experimental study	Adolescents with a Facebook account who self-identified as being between the ages of 13 and 18 years old and living in Philadelphia were targeted to receive a series of advertisements from 3forME Reach: (*n* = 155,110) Engagement: (*n* = 2107)	Vaccine uptake	The advertising campaigns did not have a strong effect on HPV vaccine uptake even when common barriers of parental consent and cost were minimized.
3.	Allen et al., (2020) [27]	To assess the feasibility and preliminary effect of a month-long Twitter campaign among a low-income and racially/ethnically diverse group of women	Quasi- experimental study	Women aged 18 to 26 years who were residents of public housing in two Massachusetts cities (*n* = 35)	Knowledge, Intention	HPV vaccination knowledge scores were low and did not change after the campaign (56% vs. 57%, *p* = 0.858) No statistically significant change in the intent to be vaccinated in the next 6 months (*p* = 1.000) or 12 months (*p* = 0.617) after the campaign among those who had not yet started or completed vaccination
4.	Kim et al., (2020) [24]	Measures how much attention audiences pay to misinformation and a correction message, and how attention is shaped by the correction strategy employed (humor versus non-humor)	Experimental	Students from a major mid- Atlantic University, over the age of 18, who could speak and understand English (*n* = 61)	Perception	Credibility ratings of the misinformation were positively associated with HPV misperceptions, while credibility ratings for the correction were negatively associated with HPV misperceptions
5.	Nan & Daily (2015) [23]	To provide insight into the polarizing effects of mixed blogs on HPV vaccine-related beliefs, including perceived vaccine efficacy and safety	Experimental	Undergraduate students with a mean age of 20 from a large East Coast university (*n* = 338)	Perception	For participants who perceived vaccines, in general, to be ineffective, exposure to the mixed blogs slightly reduced perceived efficacy of the HPV vaccine. In contrast, for participants who perceived vaccines in general to be very efficacious, exposure to the mixed blogs increased perceived efficacy of the HPV vaccine.
6.	Ortiz et al., (2018) [22]	To determine whether the strategic distribution of information about HPV and the HPV vaccine via an adolescent (ages 13–18) social media platform (i.e., Facebook) is a feasible and effective way to improve adolescents’ knowledge about the virus, vaccine, and vaccination rates.	Experimental	Adolescents with an average age of 15.6 years (SD = 1.68) participated in the final intervention (*n* = 108)	Knowledge, Attitude	Univariate tests revealed a significant within-subjects pretest to posttest difference between the four groups for knowledge gain, *F*(3, 103) = 2.76, *p* < 0.05, but not for vaccination rates, *F*(3, 103) = 1.10, *p* = 0.35. A post hoc analysis of the four groups indicated that for those participants in the intervention group who reported receiving a notification every time a new fact was posted to the Facebook page, they were significantly more likely than any other group to increase in their HPV and vaccine knowledge, *p* < 0.05.
7.	Lee & Cho (2017) [25]	Investigated whether using different message framing and media influences the public’s perceived severity/benefits/barriers, and their willingness to get vaccinated	Experimental	College students between 20 and 28 years old (M = 22.44, SD = 1.22) (*n* = 142)	Perception, Behavioral intention	No significant difference between newspaper and Facebook in their effect on behavioral intentions (*p* = 0.94). However, loss-framed messages on Facebook led to higher vaccination intentions (M = 4.90, SD = 1.33) Discovered that media channels significantly affected perceived severity and barriers to HPV vaccination. Participants exposed to online newspapers perceived greater severity of HPV-related risks compared to those exposed to Facebook (M = 5.08 vs. 4.63, *p* < 0.05)

## Data Availability

No new data were created or analyzed in this study.
